# Effects of different carbapenemase and siderophore production on cefiderocol susceptibility in *Klebsiella pneumoniae*

**DOI:** 10.1128/aac.01019-24

**Published:** 2024-10-29

**Authors:** Chengcheng Yang, Liang Wang, Jingnan Lv, Yicheng Wen, Qizhao Gao, Feinan Qian, Xiangxiang Tian, Jie Zhu, Zhichen Zhu, Liang Chen, Hong Du

**Affiliations:** 1 Department of Clinical Laboratory, The Second Affiliated Hospital of Soochow University, Suzhou, China; 2 MOE Key Laboratory of Geriatric Diseases and Immunology, The Second Affiliated Hospital of Soochow University, Suzhou, Jiangsu, China; 3 Key Laboratory of Alkene-Carbon Fibres-Based Technology and Application for Detection of Major Infectious Diseases, Suzhou, China; 4 Department of Pharmacy Practice, School of Pharmacy and Pharmaceutical Sciences, University at Buffalo, Buffalo, New York, USA; Universita degli studi di roma La Sapienza, Rome, Italy

**Keywords:** cefiderocol, drug resistance mechanism, carbapenemases, siderophores, Gram-negative bacteria (GNB), drug resistance evolution

## Abstract

The resistance mechanism of Gram-negative bacteria to the siderophore antibiotic cefiderocol is primarily attributed to carbapenemase and siderophore uptake pathways; however, specific factors and their relationships remain to be fully elucidated. Here, we constructed cefiderocol-resistant *Klebsiella pneumoniae* (CRKP) strains carrying different carbapenemases and knocked out siderophore genes to investigate the roles of various carbapenemases and siderophores in the development of cefiderocol resistance. Antimicrobial susceptibility testing revealed that both *bla*_NDM_ and *bla*_KPC_ significantly increased the minimum inhibitory concentration (MIC) of *Klebsiella pneumoniae* (KP) to cefiderocol, while *bla*_OXA-48_ showed a modest increase. Notably, KP expressing NDM exhibited a higher cefiderocol MIC compared to KP expressing KPC, although expression of NDM alone did not induce cefiderocol resistance. Laboratory evolutionary experiments demonstrated that combining pNDM with mutations in the siderophore uptake receptor gene *cirA* and pKPC with a mutation in the two-component system gene *envZ* led to KP reaching a high level of cefiderocol resistance. Although combining pOXA with mutations in the two-component system gene *baeS* did not induce cefiderocol resistance, it significantly reduced susceptibility. Moreover, siderophores could influence the development of cefiderocol resistance. Strains deficient in enterobactin exhibited increased susceptibility to cefiderocol, while deficiencies in yersiniabactin and salmochelin showed no significant alterations. In conclusion, carbapenemase gene expression facilitates cefiderocol resistance, but its presence alone is insufficient. Cefiderocol resistance in CRKP typically involves abnormal expression of certain genes and other factors, such as mutations in siderophore uptake receptor genes and two-component system genes. The enterobactin siderophore synthesis gene *entB* may also contribute to resistance.

## INTRODUCTION

Antibiotic resistance poses a global health challenge that exerts significant pressure on healthcare systems (1, 2). β-Lactam drugs are the cornerstone of antibiotic therapy and are widely used to treat Gram-negative bacterial (GNB) infections. Carbapenems are the most potent β-lactam drugs for treating bacterial infections, particularly those caused by Enterobacterales (3). However, since their discovery in the 1990s, carbapenem-resistant Enterobacterales (CRE) has rapidly spread globally, posing a serious threat to human health (4, 5). In 2024, the World Health Organization listed CRE as one of the critical priority pathogens, highlighting the urgent need for the development of new antibiotics (6).

The recently introduced novel cephalosporin, cefiderocol, represents a promising treatment option for infections caused by multidrug resistant and carbapenemase-producing GNB. Cefiderocol, a new class of antibiotics formed by covalently linking a siderophore molecule to a chemical moiety with antimicrobial properties (7), has been approved in the European Union and the United States for the treatment of GNB infections in adults (8). Cefiderocol exhibits superior microbiological activity against GNB compared to ceftazidime-avibactam (AVI) and meropenem. Its cephalosporin backbone is linked to an iron-chelating group, allowing it to enter the bacterial periplasm via the bacterial iron transport system. This “Trojan horse strategy” helps it evade porin-mediated resistance mechanisms. Additionally, the ceftazidime- and cefepime-related side chains of cefiderocol enhance hydrolytic stability against various β-lactamases (9). Although the current isolation rate of clinically resistant strains to cefiderocol remains low, reports of cefiderocol resistance have emerged (10, 11). A thorough investigation into the potential mechanisms of cefiderocol resistance is essential for providing insights to prevent its development and may inspire the creation of new antibiotics. It is now widely believed that resistance to cefiderocol is associated with mutations in siderophore uptake receptors (12, 13). Furthermore, the presence of various β-lactamases (such as PER, SHV, BEL, and NDM types) has also been shown to influence bacterial resistance to cefiderocol (14).

However, the understanding of potential resistance factors and mechanisms related to cefiderocol remains inadequate, significantly hindering its clinical application. To address this gap, we engineered a *Klebsiella pneumoniae* (KP) strain containing various carbapenemase genes and conducted *in vitro* selection experiments under cefiderocol pressure. This approach enabled us to investigate the effects of different carbapenemases on cefiderocol resistance. Additionally, we constructed siderophore-deficient strains to examine the impact of siderophore production on cefiderocol susceptibility.

## RESULTS

### Carbapenemase-mediated changes in cefiderocol susceptibility

**Fig 1:**
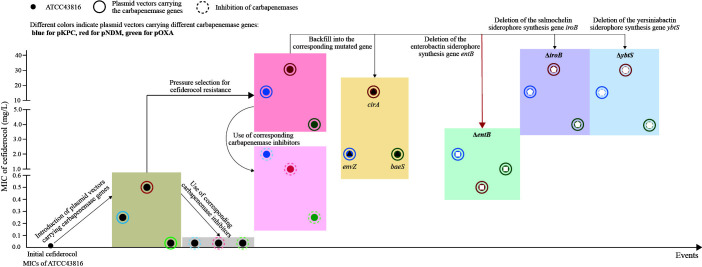
Changes in the cefiderocol MICs of strains at critical points during the experimental process. The vertical axis represents the cefiderocol MICs, while the horizontal axis represents the occurrence of events throughout the progression of the experiment.

To assess the impact of different carbapenemases on cefiderocol resistance, we introduced the three most common carbapenemase genes (*bla*_KPC_, *bla*_NDM_, and *bla*_OXA-48_) into the same genetic background of *K. pneumoniae* ATCC 43816 using plasmid vectors. ATCC 43816 was the first hypervirulent strain of *Klebsiella pneumoniae* (hvKP) discovered, isolated from a patient with a liver abscess in Hong Kong. Initial MICs of cefiderocol were then determined. As listed in Table 1 and illustrated in Fig. 1, cefiderocol MICs were markedly elevated (>16-fold and>32-fold) for strains carrying *bla*_KPC-2_ and *bla*_NDM-1_ vectors (pKPC and pNDM) compared to the wild-type strain ATCC 43816. However, the presence of the *bla*_OXA-48_ vector (pOXA) resulted in only a modest change in cefiderocol susceptibility (twofold). *bla*_NDM-1_ induced a greater increase in cefiderocol MIC compared to *bla*_KPC-2_ and *bla*_OXA-48_, although it did not reach the threshold for cefiderocol resistance. For Enterobacterales, both the Clinical and Laboratory Standards Institute (CLSI) and the Food and Drug Administration consider cefiderocol MIC values of ≤4 mg/L to be susceptible and ≥16 mg/L to be resistant (15). The European Committee on Antimicrobial Susceptibility Testing considers cefiderocol MIC values of≤2 mg/L to be sensitive and >2 mg/L to be resistant (16).

**TABLE 1:** Initial cefiderocol MICs of hvKP expressing different β-lactamases

MIC (mg/L）	Strains			
	ATCC 43816	pKPC	pNDM	pOXA-48
Cefiderocol	<0.015	0.25	0.5	0.03

### Metallo**-**β-lactamases (NDM) confer higher resistance potential to cefiderocol than serine-β-lactamases (KPC and OXA-48), but neither can alone cause cefiderocol resistance

**Fig 2:**
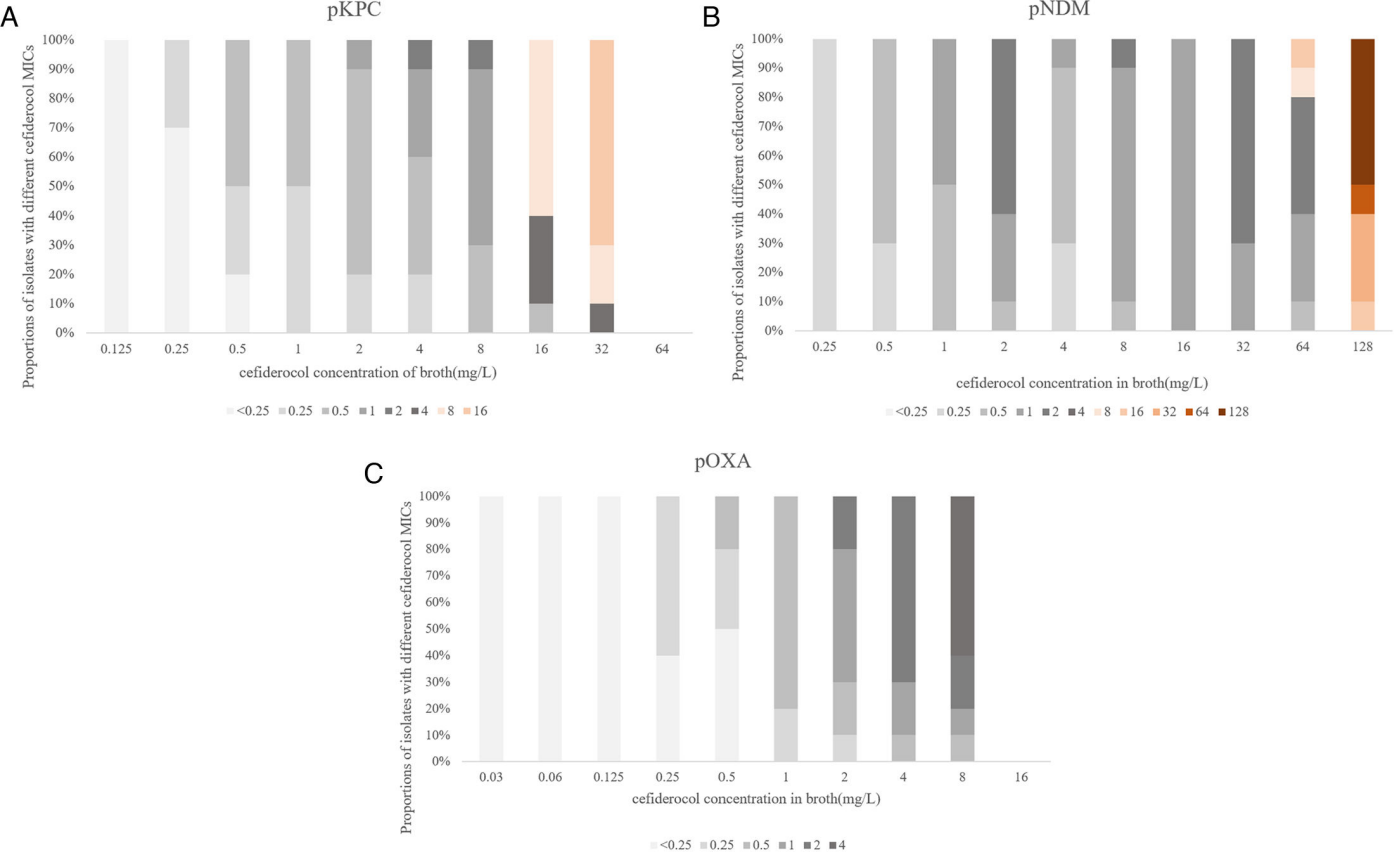
MICs and proportions of 10 randomly picked colonies in each generation (panels A, B, and C represent pKPC, pNDM, and pOXA, respectively). The vertical axis represents the proportions of isolates with different cefiderocol MICs, while the horizontal axis represents the cefiderocol concentrations used in the serial passage experiment. Different colors indicate the MICs of each colony.

To further investigate the effects of β-lactamases and other potential resistance factors on cefiderocol resistance, we performed *in vitro* cefiderocol selection experiments on strains carrying pKPC, pNDM, and pOXA, respectively. During the selection process, growth ceased for the pKPC and pOXA strains at cefiderocol concentrations of 64 and 16 mg/L, respectively, while the pNDM strain continued to grow until the concentration reached 128 mg/L. A 5-µL culture from each induced concentration was plated on lysogeny broth plates, and 10 colonies were randomly selected for cefiderocol MIC testing (as depicted in Fig. 2). The MICs of the 10 randomly selected colonies increased proportionally with the escalating cefiderocol concentrations, although they did not reach the exact concentrations present in the cultures. Notably, resistant strains of pKPC and pNDM (MIC >4 mg/L) were isolated at the beginning of the subculture step at cefiderocol concentrations of 16 and 64 mg/L, respectively. However, pOXA did not yield any resistant colonies throughout the selection process. Ultimately, the isolates demonstrating the highest stable resistance to cefiderocol were designated as pKPC-r (MIC = 16 mg/L), pNDM-r (MIC = 32 mg/L), and pOXA-r (MIC = 4 mg/L), respectively (as shown in Table 2; Fig. 1). Consistent with the pre-selection data, the NDM-producing strain (pNDM-r) retained the highest cefiderocol MIC after cefiderocol resistance selection. These results suggested that, compared to the serine β-lactamases KPC and OXA, the metallo-β-lactamase NDM exhibited a greater potential for cefiderocol resistance during selection.

**TABLE 2:** Cefiderocol MICs of hvKP expressing different β-lactamases^*a*^

MIC (mg/L）	Strains					
	pNDM	pNDM-r	pKPC	pKPC-r	pOXA	pOXA-r
Cefiderocol	0.5	32	0.25	16	0.03	4
Cefiderocol + AVI			0.03	2	0.03	0.25
Cefiderocol + DPA	0.03	1				

^*a*^ AVI, avibactam; DPA, 2,6-dipicolinic acid.

To understand the mechanism associated with cefiderocol resistance, we first performed Sanger sequencing on the three carbapenemase genes in pKPC-r, pNDM-r, and pOXA-r. The results showed that none of the *bla*_KPC-2_, *bla*_NDM-1_, or *bla*_OXA-48_ genes were mutated. We then examined whether cefiderocol resistance was independent of the presence of these carbapenemase genes. To this end, we conducted antimicrobial susceptibility testing (AST) with and without the β-lactamase inhibitors AVI (for pKPC-r and pOXA-r) and dipicolinic acid (DPA) (for pNDM-r). These inhibitors target serine-β-lactamases and metallo-β-lactamases, respectively (Table 2; Fig. 1). The MICs of cefiderocol were significantly reduced by the addition of the inhibitors, indicating that β-lactamases play a role in cefiderocol resistance. Specifically, the addition of AVI reduced the MICs of pKPC-r and pOXA-r by 8-fold (from 16 to 2 mg/L) and 16-fold (from 4.0 to 0.25 mg/L), respectively. Meanwhile, DPA reduced the MIC of pNDM-r by 64-fold (from 32 to 0.5 mg/L). Although none of the β-lactamase inhibitors restored the corresponding cefiderocol MIC to the initial level, the MICs of pKPC-r, pNDM-r, and pOXA-r remained significantly higher than those of pKPC, pNDM, and pOXA. This finding suggests that additional factors may contribute to the emergence of cefiderocol-resistant strains.

### Carbapenemase combined with abnormal expression of siderophore uptake receptor *cirA*, two-component system *envZ*, and *baeS* can cause cefiderorol resistance

The constructed pKPC, pNDM, and pOXA strains, along with cefiderocol-resistant pKPC-r, pNDM-r, and pOXA-r strains, were subjected to next-generation sequencing. Comparative genomic analysis revealed that the strains with reduced cefiderocol susceptibility (pKPC-r, pNDM-r, and pOXA-r) all harbored mutations in a single gene compared to the pKPC, pNDM, and pOXA strains (Table 3; Fig. 1). The gene mutated in pKPC-r was the two-component system gene *envZ*, with all three sequenced resistant strains carrying the same missense mutation, T434G. It was previously thought that certain missense alleles of *envZ* (encoding the kinase of the EnvZ/OmpR two-component system) negatively affected the expression of iron uptake genes. However, data from Gerken et al. (17) indicated that constitutive *envZ* alleles activated the Feo- and OmpC-mediated iron uptake pathways, flooding the cytoplasm with available ferrous iron. This activation triggered the ferric uptake regulator, Fur, which inhibited the ferric uptake system but did not inhibit the *feo* operon due to the positive effect of activated EnvZ/OmpR (17). The catecholate siderophore receptor gene *cirA* in three pNDM-r strains was mutated, including two with premature stop codons (C879A and G772A) and one with a frameshift mutation (462_463insT). The two-component system gene *baeS* in pOXA-r was mutated, as well as all three pOXA-r strains containing missense mutations at T598G and C541T. Previous studies have shown that certain mutations in *baeS* (encoding the baeS/baeR two-component system kinase) can lead to increased efflux pump activity, resulting in resistance to multiple antibiotics, including cefiderocol (18, 19).

**TABLE 3:** Mutated genes of cefiderocol-resistant strains

Strains	Mutated genes	Sites		Description
pKPC-r	*envZ*	T434G	NZ_CP009208.1. Val145Gly	Missense mutation
pKPC-r2		T434G	NZ_CP009208.1. Val145Gly	Missense mutation
pKPC-r3		T434G	NZ_CP009208.1. Val145Gly	Missense mutation
pNDM-r	*cirA*	C879A	NZ_CP009208.1. Tyr293*	Early termination
pNDM-r2		462_463insT	NZ_CP009208.1. Ile155Tyrfs	Point insert
pNDM-r3		C772T	NZ_CP009208.1. Gln258*	Early termination
pOXA-r	*baeS*	T598G	NZ_CP009208.1. Thr200Gly	Missense mutation
pOXA-r2		C541T	NZ_CP009208.1 Asp181Asn	Missense mutation
pOXA-r3		C541T	NZ_CP009208.1 Asp181Asn	Missense mutation

To investigate whether the above genes play a role in the reduction of cefiderocol sensitivity, we constructed cloning vectors with wild-type *envZ*, *cirA*, and *baeS* genes and introduced them into the corresponding resistant mutant strains. The results of cefiderocol susceptibility testing (Table 4) indicated that all cefiderocol MICs were reduced after complementation with the corresponding wild-type genes compared to the selected resistant strains. This finding suggests that *envZ*, *cirA*, and *baeS* genes all contribute to the process of cefiderocol resistance.

**TABLE 4:** Cefiderocol MICs of hvKP expressing different β-lactamases

MIC (mg/L）	Strains					
	pNDM-r	pNDM-r-pBAD33-*cirA*	pKPC-r	pKPC-r-pBAD33-*envZ*	pOXA-r	pOXA-r- pBAD33-*baeS*
Cefiderocol	32	16	16	2	4	2

### Deletion of the enterobactin siderophore synthesis gene *entB* results in a substantial increase in cefiderocol susceptibility

To explore the effects of different siderophores on cefiderocol sensitivity, we generated mutants deficient in enterobactin and salmochelin (a glycosylated variant of enterobactin) (Δ*entB*), salmochelin (Δ*iroB*), and yersiniabactin (Δ*ybtS*) in pNDM-r, pKPC-r, and pOXA-r (the standard strain ATCC 43816 used in this study secretes only these three types of siderophores). Interestingly, strains lacking both enterobactin and salmochelin (Δ*entB*) exhibited the most significant reduction in cefiderocol MICs, with pNDM-rΔ*entB* showing a >32-fold decrease compared to pNDM-r with wild-type *entB*. Meanwhile, pKPC-rΔ*entB* and pOXA-rΔ*entB* demonstrated a modest fourfold decrease. Complementation experiments further confirmed these findings, as restoring *entB* partially restored the MICs of cefiderocol compared to the mutants. Additionally, overexpression of *entB* in pNDM led to a mild increase in MICs, although there was no significant change for pKPC or pOXA. In contrast, strains deficient in salmochelin or yersiniabactin alone showed no discernible difference in cefiderocol MICs (Table 5; Fig. 1). This led us to conclude that enterobactin, a catecholate siderophore, contributes to cefiderocol resistance, possibly due to competition between enterobactin and cefiderocol for Fe(III) or their respective receptors.

**TABLE 5:** Antimicrobial drug susceptibility profile

MIC (mg/L）	Strains								
	pNDM	pNDM-r	pNDM-r Δ*entB*	pNDM-r Δ*iroB*	pNDM-r Δ*ybtS*	pNDM-r/*entB*	pNDM-r Δ*entB*/*entB*	pNDM-r Δ*iroB*/*iroB*	pNDM-r Δ*ybtS*/*ybtS*
Cefiderocol	0.5	32	0.5	32	32	64	16	32	32
Cefiderocol + DPA	0.03	1	0.0625	5	0.5				

MIC (mg/L）	Strains								
	pKPC	pKPC-r	pKPC-r Δ*entB*	pKPC-r Δ*iroB*	pKPC-r Δ*ybtS*	pKPC-r/*entB*	pKPC-r Δ*entB*/*entB*	pKPC-r Δ*iroB*/*iroB*	pKPC-r Δ*ybtS*/*ybtS*
Cefiderocol	0.25	16	2	16	16	32	16	16	16
Cefiderocol + AVI	0.03	2	0.25	2	2				

MIC (mg/L）	Strains								
	pOXA	pOXA-r	pOXA-r Δ*entB*	pOXA-r Δ*iroB*	pOXA-r Δ*ybtS*	pOXA-r/*entB*	pOXA-r Δ*entB*/*entB*	pOXA-r Δ*iroB*/*iroB*	pOXA-r Δ*ybtS*/*ybtS*
Cefiderocol	0.03	4	1	4	4	2	4	4	4
Cefiderocol + AVI	0.03	0.25	0.06	0.25	0.25				

## DISCUSSION

Surveillance data indicate that *K. pneumoniae* strains producing NDM generally exhibit higher MICs for cefiderocol (20). In our current study, we observed a similar trend through laboratory experiments: the cefiderocol MIC of pNDM was notably higher compared to that of pKPC and pOXA. This underscores that NDM, as a metallo-β-lactamase, confers elevated levels of antimicrobial resistance to cefiderocol compared to serine-β-lactamases within the same genetic context. The mechanism by which different carbapenemase enzymes influence cefiderocol susceptibility to varying extents remains incompletely understood, and we are conducting further studies on this. Importantly, while the presence of pKPC, pNDM, and pOXA-48 resulted in an increase in cefiderocol MIC, it did not reach the resistance breakpoint. This suggests that carbapenemase expression alone is insufficient to induce cefiderocol resistance (10, 21). Furthermore, throughout laboratory evolution experiments, pNDM consistently demonstrated a higher potential for cefiderocol resistance compared to pKPC and pOXA. However, the cefiderocol MIC for pOXA-r did not increase to the same degree, indicating a nuanced response. Therefore, caution is advised when using cefiderocol to treat bacteria harboring *bla*_NDM_ to mitigate the risk of resistance emergence.

We also investigated other potential factors that may contribute to cefiderocol resistance in conjunction with carbapenemases. Through our study on resistance selection, we identified three key genes implicated in cefiderocol resistance. Among these, the siderophore uptake receptor *cirA* has previously been linked to cefiderocol resistance (12, 22). Our preliminary findings suggest that both the *envZ* and *baeS* genes of the two-component system also play a role in conferring cefiderocol resistance, as demonstrated through vector complementation experiments. Additionally, the use of corresponding carbapenemase inhibitors can restore the susceptibility of cefiderocol-resistant strains to cefiderocol. This suggests that combining carbapenemase inhibitors with cefiderocol may hinder the development of cefiderocol-resistant CRE strains, significantly enhancing treatment efficacy.

Iron plays a crucial role in various essential biological processes such as electron transfer, amino acid synthesis, DNA synthesis, and protection against superoxide free radicals. For microorganisms, acquiring iron from the environment—especially during infection when competing with the host—is imperative for survival. Consequently, microorganisms have evolved intricate iron uptake systems, with the siderophore uptake system being paramount among Gram-negative bacteria.

Cefiderocol is a new antibacterial agent that can chelate with Fe(III). Its unique feature lies in its ability to utilize the siderophore uptake system to penetrate the bacterial periplasm, thus circumventing the influence of efflux pumps and porins to a certain extent, resulting in potent bactericidal effects. Previous studies have underscored the significant impact of siderophore uptake receptors on cefiderocol sensitivity (23).

The siderophore uptake system comprises siderophores, siderophore uptake receptors, ABC transporters, energy supply systems, and other components. However, apart from receptors, limited research has explored the relationship between other system components and cefiderocol resistance, particularly concerning different siderophores. Siderophores are low-molecular-weight organic compounds with a specific affinity for Fe(III) and are indispensable for microorganisms in acquiring, transporting, and utilizing iron. They are classified into catecholate, phenolate, hydroxamate, and mixed types (24, 25). *K. pneumoniae* produces four siderophores—enterobactin, salmochelin, yersiniabactin, and aerobactin—to fulfill its iron requirements for essential processes. Among these, enterobactin, a prototype of catecholate siderophores, excels at competing with host iron-binding proteins for iron. In response to bacterial infections, host cells secrete lipocalin2 (Lcn2) as a defense mechanism (26). To evade Lcn2, bacteria have evolved salmochelin, a glycosylated variant of enterobactin (27). Yersiniabactin, encoded within a pathogenicity island, represents a siderophore-dependent iron uptake system prevalent among pathogenic bacteria (28). In contrast, aerobactin primarily confers virulence to hypervirulent *K. pneumoniae* strains, facilitating systemic infections (29).

Siderophore uptake receptors play a pivotal role in the evolution of cefiderocol resistance, as siderophores and their receptors are integral to iron uptake. Consequently, it remains unclear whether different siderophores also influence cefiderocol resistance. Therefore, we investigated the impact of three key genes involved in siderophore synthesis (*entB*, *iroB*, and *ybtS*) on cefiderocol sensitivity. Our results indicate for the first time that deleting *entB* significantly reduces cefiderocol’s MIC, while deleting *iroB* and *ybtS* has no significant effect. This may suggest that cefiderocol and the enterobactin siderophore share similarities in their corresponding receptor-binding sites. Constructing antibiotics with salmochelin and yersiniabactin receptor-binding sites, combined with cefiderocol, may offer a more effective antibiotic treatment. Ultimately, this finding enhances our understanding of cefiderocol’s resistance mechanism, aiding in resistance prevention and providing a theoretical foundation for promoting and developing cefiderocol and novel siderophore antibiotics.

In summary, our study reveals that various types of carbapenemases can contribute to cefiderocol resistance to different extents, but carbapenemase expression alone is insufficient to confer this resistance. The presence of other genetic features, including defects in siderophore uptake receptors and two-component systems, also plays a significant role. Importantly, our results demonstrate that among the three siderophores produced by *K. pneumoniae*, the production of enterobactin is closely linked to cefiderocol activity. These findings illuminate the multifaceted mechanisms underlying cefiderocol resistance in *K. pneumoniae*.

## MATERIALS AND METHODS

### Bacterial strains

The ST439 K2 hypervirulent *K. pneumoniae* ATCC 43816 was obtained from the ATCC and used as the wild-type strain. Given reports of reduced cefiderocol efficacy against serine-β-lactamases (30, 31) and metallo-β-lactamases (32), we cloned *bla*_KPC-2_, *bla*_NDM-1_, and *bla*_OXA-48_ into pET28a-rpsL vector, creating strains with an identical genetic background with different carbapenemase genes: ATCC 43816 pET28a-rpsL-KPC (pKPC), ATCC 43816 pET28a-rpsL-NDM (pNDM), and ATCC 43816 pET28a-rpsL-OXA (pOXA). To ensure continuous gene expression and enhance β-lactam hydrolysis, we integrated the rpsL promoter from pCasKP into the pET28a plasmid. The strains used in this experiment are listed in Table 6.

**TABLE 6:** Description of the strains mentioned in this experiment

Strains	Description	β-Lactamase	Origin
ATCC 43816	Wild type	None	Type strain
pKPC	ATCC 43816 transformed with pET28a-*rpsL*-KPC	KPC	This study
pNDM	ATCC 43816 transformed with pET28a-*rpsL*-NDM	NDM	This study
pOXA	ATCC 43816 transformed with pET28a-*rpsL*-OXA	OXA-48	This study
pKPC-r	pKPC and evolved resistant to cefiderocol	KPC	This study
pNDM-r	pNDM and evolved resistant to cefiderocol	NDM	This study
pOXA-r	pOXA and evolved resistant to cefiderocol	OXA-48	This study
pKPC-rΔ*entB*	pKPC-r deficient in *entB*	KPC	This study
pKPC-rΔ*iroB*	pKPC-r deficient in *iroB*	KPC	This study
pKPC-rΔ*ybtS*	pKPC-r deficient in *ybtS*	KPC	This study
pNDM-rΔ*entB*	pNDM-r deficient in *entB*	NDM	This study
pNDM-rΔ*iroB*	pNDM-r deficient in *iroB*	NDM	This study
pNDM-rΔ*ybtS*	pNDM-r deficient in *ybtS*	NDM	This study
pOXA-rΔ*entB*	pOXA-r deficient in *entB*	OXA-48	This study
pOXA-rΔ*iroB*	pOXA-r deficient in *iroB*	OXA-48	This study
pOXA-rΔ*ybtS*	pOXA-r deficient in *ybtS*	OXA-48	This study
pKPC-r/*entB*	pKPC-r transformed with pBAD33-*entB*	KPC	This study
pKPC-rΔ*entB*/*entB*	pKPC-rΔ*entB* transformed with pBAD33-*entB*	KPC	This study
pKPC-rΔ*iroB*/*iroB*	pKPC-rΔ*iroB* transformed with pBAD33-*iroB*	KPC	This study
pKPC-rΔ*ybtS*/*ybtS*	pKPC-rΔ*ybtS* transformed with pBAD33-*ybtS*	KPC	This study
pNDM-r/*entB*	pNDM-r transformed with pBAD33-*entB*	NDM	This study
pNDM-rΔ*entB*/*entB*	pNDM-rΔ*entB* transformed with pBAD33-*entB*	NDM	This study
pNDM-rΔ*iroB*/*iroB*	pNDM-rΔ*iroB* transformed with pBAD33-*iroB*	NDM	This study
pNDM-rΔ*ybtS*/*ybtS*	pNDM-rΔ*ybtS* transformed with pBAD33-*ybtS*	NDM	This study
pOXA-r/*entB*	pOXA-r transformed with pBAD33-*entB*	OXA-48	This study
pOXA-rΔ*entB*/*entB*	pOXA-rΔ*entB* transformed with pBAD33-*entB*	OXA-48	This study
pOXA-rΔ*iroB*/*iroB*	pOXA-rΔ*iroB* transformed with pBAD33-*iroB*	OXA-48	this study
pOXA-rΔ*ybtS*/*ybtS*	pOXA-rΔ*ybtS* transformed with pBAD33-*ybtS*	OXA-48	this study

### Antimicrobial susceptibility test

We conducted AST using the standard broth microdilution method in accordance with CLSI guidelines (33). The determination of the MIC for cefiderocol followed the procedure outlined by Hackel et al., utilizing iron-depleted cation-adjusted Mueller-Hinton broth (ID-CAMHB) (34). All tests were repeated in triplicate.

### Laboratory evolutionary experiments

We initially determined the cefiderocol MIC for the three constructs: pKPC, pNDM, and pOXA. To obtain cefiderocol-resistant strains, we conducted a serial passage experiment involving increasing concentrations of cefiderocol. Briefly, we started with a colony grown on a lysogeny broth agar (LBA) plate, which was then cultured in 5 mL of ID-CAMHB containing cefiderocol at an initial sublethal inhibitory concentration (half of the MIC). This culture was maintained at 37°C with constant shaking at 200 rpm for 18 hours. The following day, we transferred the overnight culture by inoculating 50 µL into 5 mL (1:100) of fresh medium, with antibiotic concentrations continually doubling until either no growth was observed or a final concentration of 128 mg/L was reached. Simultaneously, each subculture was inoculated onto LBA plates containing 50-mg/L kanamycin and incubated at 37°C for 24 hours. Distinct colonies were randomly selected to determine their cefiderocol MIC using ID-CAMHB. Finally, the colonies with the highest MIC were inoculated onto LBA plates containing 50-mg/L kanamycin for serial passage over 10 days. This process aimed to select colonies in which drug resistance phenotypes were stably maintained while the genetic background underwent alterations (35, 36).

### Construction of the *entB*, *iroB*, and *ybtS* mutants

The *entB*, *iroB*, and *ybtS* mutants of pKPC-r, pNDM-r, and pOXA-r were created using the CRISPR/Cas9-mediated genome-editing system (37). First, we introduced the plasmid pCasKP into pKPC-r, pNDM-r, and pOXA-r through electroporation, followed by selection on agar plates containing 30-mg/L apramycin. Successful transformation was confirmed using PCR. The 20-bp targeting region (N20) of the sgRNA was designed using the online tool available at http://crispr.dfci.harvard.edu/SSC/. Linear homologous DNA fragments were used as repair templates. The repair templates, along with pSGKP-Rif-*entB*/*iroB*/*ybtS*-N20, were co-transformed into L-arabinose-induced, pCasKP-positive pKPC-r, pNDM-r, and pOXA-r strains to facilitate *entB*, *iroB*, and *ybtS* gene deletions. Cultures were then plated on LBA plates containing 30-mg/L apramycin and 100-mg/L rifampicin. We confirmed the mutants by conducting PCR and Sanger sequencing on selected colonies.

### Complement of the *entB*, *iroB*, and *ybtS* mutants and overexpression of *entB*

For the complementation experiment, the amplified DNA fragment was ligated into pBAD33 and subsequently transformed into *Escherichia coli* DH5α. Selection was carried out on LBA plates supplemented with chloramphenicol (100 mg/L). The resulting recombinant plasmid was then extracted and introduced into the mutant strains. Verification of the complementation strains was conducted by performing PCR and Sanger sequencing on selected colonies. The expression plasmid pBAD33, induced with L-arabinose, was grown in the presence of 0.2% arabinose.
